# Hot Isostatic Pressing for Fatigue Critical Additively Manufactured Ti-6Al-4V

**DOI:** 10.3390/ma15062051

**Published:** 2022-03-10

**Authors:** Terrence P. Moran, Patricio E. Carrion, Seungjong Lee, Nima Shamsaei, Nam Phan, Derek H. Warner

**Affiliations:** 1 Cornell Fracture Group, Sibley School of Mechanical and Aerospace Engineering, Cornell University, Ithaca, NY 14853, USA; tpm92@cornell.edu; 2 National Center for Additive Manufacturing Excellence (NCAME), Auburn University, Auburn, AL 36849, USA; pec0016@auburn.edu (P.E.C.); szl0169@auburn.edu (S.L.); shamsaei@auburn.edu (N.S.); 3 Department of Mechanical Engineering, Auburn University, Auburn, AL 36849, USA; 4 Structures Division, Naval Air Systems Command, Patuxent River, MD 20670, USA; nam.phan@navy.mil; 5 Cornell Fracture Group, School of Civil and Environmental Engineering, Cornell University, Ithaca, NY 14853, USA

**Keywords:** metal additive manufacturing, fatigue, hot isostatic pressing, Ti-6Al-4V

## Abstract

The efficacy of hot isostatic pressing (HIP) for enhancing fatigue performance is investigated for additively manufactured (AM) Ti-6Al-4V. The limitations of HIP are probed by varying the initial material state via the selection of AM system, powder chemical composition, and process parameters. We demonstrate that the fatigue performance of HIP’d AM Ti-6Al-4V depends on the as-built quality of the material. Differences in common material attributes, such as pre-HIP defect populations or post-HIP microstructure morphology, are shown to be insufficient to explain the observed discrepancies in performance. This implies that additional microstructure attributes or localized deviations from the expected structure control the failure of this material. Finally, HIP parameters outside ASTM recommendations were explored, where a reduced temperature and high-pressure treatment yielded significantly improved fatigue performance.

## 1. Introduction

Ti-6Al-4V is an α+β titanium alloy with an excellent combination of specific strength, corrosion resistance, fracture toughness, and biocompatibility [1,2,3]. First developed in the 1950s for the construction of aircraft components, demand for Ti-6Al-4V is still dominated by the aerospace industry [4]. More recently, this alloy has also been used for other applications, such as biomedical implants [2]. The utilization of additive manufacturing (AM) technology for fatigue critical applications of Ti-6Al-4V has been a long sought goal [5,6,7]. Due to the unique thermal history experienced by the material during AM fabrication, a disparate set of material properties are observed in AM Ti-6Al-4V when compared to traditional fabrication methods [8]. For this popular structural alloy, controlling the formation of microscopic build defects during fabrication can be key towards the goal of reliable fatigue performance [9]. Despite significant progress to better understand, predict, improve, and monitor the AM process, the control of build defects is not yet sufficient to assure safety in demanding fatigue critical applications [10,11,12,13,14,15,16,17,18,19,20]. In such cases, post processing treatments, such as hot isostatic pressing (HIP) followed by surface machining, have been used in an effort to minimize the negative implications of build defects [21,22,23].

Early studies indicated that HIP treatment of AM Ti-6Al-4V was capable of delivering fully dense parts by completely closing subsurface build defects [24,25,26]. Subsequent work refined this view, demonstrating that internal defects are not fully closed by HIP treatment, due to the insolubility of argon shielding gas and the subsequent build-up of an internal pore pressure [7,27,28]. Nonetheless, HIP has continually demonstrated an ability to minimize or eliminate the adverse effects of internal build defects on fatigue behavior, providing performance comparable to traditionally fabricated wrought material [22,23,29,30]. The performance gains offered by HIP have been attributed to two distinct mechanisms [22]. While not fully closing all internal defects, HIP treatment does reduce the size of defects across the population. This reduces the number of defects with sizes above the critical threshold required to initiate a fatigue crack. Second, while reducing the size of defects, the local microstructure surrounding the defects is refined, inhibiting the formation of life limiting fatigue cracks.

Fatigue data sets from independent research groups provide compelling evidence that HIP consistently produces AM Ti-6Al-4V fatigue performance comparable to traditional wrought material, independent of initial defect population attributes (i.e., defect type, size, and distribution before HIP) [7,22,31,32]. These studies report that after HIP, the fatigue life of AM Ti-6Al-4V is governed by crack initiation from microstructural features, not build defects [7,27,29,32,33,34]. This assertion that HIP can enhance fatigue performance independent of the initial defect population has substantial implications for the design of fatigue critical AM components, as limits to this enhancement must exist. Specifically, overestimation of the expected fatigue life after HIP has the potential for catastrophic failure in fracture critical applications of AM components, and is thus deserving of careful examination.

In this study the efficacy and limitations of HIP treatment on AM Ti-6Al-4V are investigated, with potential correlations between material attributes and fatigue performance explored. First, the independence of defects and surface finished AM Ti-6Al-4V fatigue performance after ASTM standard HIP treatment is tested. Next, the efficacy of this HIP treatment to improve the fatigue performance of specimens with variable initial material states is explored. Finally, in an attempt to improve fatigue performance, alternatives to the ASTM recommended HIP parameters are investigated. Fatigue tests were performed on five unique sets (8-10 specimens per set) of HIP’d Ti-6Al-4V specimens having varying initial material states. Before HIP treatment, defect populations were quantified via X-ray computed tomography (CT). After HIP treatment, all specimens were machined to the same hourglass geometry and surface finished, removing surface roughness and near surface defects not minimized by HIP treatment. The post-HIP microstructure morphologies and fracture surfaces were examined via electron backscatter diffraction analysis (EBSD) and optical microscopy, respectively. Additional details on these experimental procedures are presented in the following section.

## 2. Material and Methods

### 2.1. Specimen Fabrication

Two common commercial LPBF machines, the EOS M290 (EOS GmbH, Krailling, Germany) and the ProX DMP 320 (3D systems, Rock Hill, SC, USA) were used to fabricate Ti-6Al-4V specimens. These specimens were divided into five groups. Groups SH-L and SH-H were fabricated with the M290 (EOS GmbH, Krailling, Germany) which employs a 400 W Yb-fiber laser with a 1100 nm wavelength using a F-θ lens. Both groups consisted of 10 specimens. Specimens had a square cross-section and were rotated 45${}^{\circ }$ about the build axis, with an edge length of 14 mm and a height of 105 mm. The cuboid specimens were fabricated using grade 23 Ti-6Al-4V powder stock from LPW Technology Ltd, Runcorn, UK. Reused powder was utilized during fabrication as fine particle agglomerates, which are more common in virgin powders, have been shown to increase defect populations and reduce the mechanical response of LPBF components [6,35]. Specimens were distributed across the 625 mm${}^{2}$ build plate surface in accordance with NASA recommendations [36]. Group SH-L utilized the M290 manufacturer recommended default build parameters for Ti-6Al-4V. Group SH-H was fabricated with reduced infill laser power to intentionally fabricate low quality, high defect density components. These parameters are presented in Table 1. Groups SH-M, SB-M, and LTHP-M were fabricated on a DMP 320, which utilizes a 500 W Yb-fiber laser with a 1070 nm wavelength. The infill build parameters followed a bi-directional parallel scanning strategy and are outlined in Table 1. Each group contained eight specimens, which were fabricated with reused Tekmat ASTM grade 23 Ti-6Al-4V powder stock (KBM Advanced Materials, Fairfield, OH, USA). Specimens had an edge length of 13 mm, with a length of 94mm along their long axis. After fabrication, specimens were removed from the build plate via electrical discharge machining.

### 2.2. X-ray Computed Tomography

**Figure 1:**
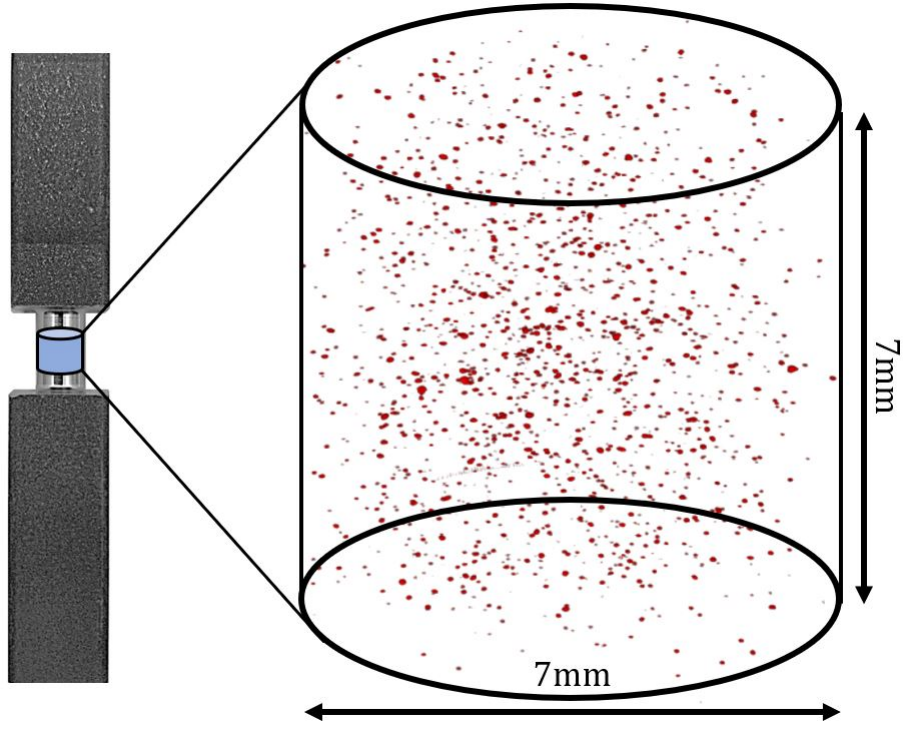
Defect population distribution measured via X-ray CT of a specimen containing a high defect density before undergoing standard HIP [18]. Note: the midsection of specimens were reduced prior to scanning for improved scan time and noise reduction.

Before specimens underwent HIP treatment, the initial defect populations were quantified. X-ray computed tomography was performed on a Versa 520 micro-CT (Zeiss, Oberkochen, Germany). A cylinder with height and diameter of 7 mm was scanned at the midpoint of each specimen’s long axis as seen in Figure 1. To reduce scan time and noise due to excess material, the center of each specimen’s cross-section was reduced along the mid section. X-ray CT was performed on the reduced section with the following parameters: power of 10 W, current of 71.6 μA, and an acceleration voltage of 140 kV. 1601 projections were collected on a scintillator detector rotating 360${}^{\circ }$ around the specimen axis with 2.5–5 s exposure times for each scan. Voxel sizes were maintained at about 7 μm, entailing approximately 785 M voxels per scan. 3D analysis of the CT data was performed via the software package ImageJ [37], where low-density regions containing eight or more contiguous 7 μm voxels were classified as build defects (an effective spherical diameter of 17 μm). For reference, defects below a critical size of ∼26 μm are thought to be inert with respect to their contribution to high cycle fatigue failures in LPBF T-6Al-4V [27]. However, as a result of the relatively small defects found in HIP’d LPBF Ti-6Al-4V, fatigue crack initiation is not governed by the largest or most geometrically detrimental defect, as expected with traditional fracture mechanics [22,27,38]. For these reasons, the shape and size of the largest defects in each specimen are not considered in this work.

Defect populations in each scanned specimen were analyzed using three metrics. The first was defect density, or the number of defects meeting the criteria defined above per unit of scanned volume. The second measurement was porosity, calculated by taking the ratio of the total defect volume to total scanned volume. The final metric was the effective diameter of individual defects. For each cluster of contiguous low-density voxels within the scanned section meeting the build defect criteria defined above, an effective defect diameter was calculated by assuming the shape of these low density voxels’ volume to be spherical. Our recent work has shown that systemic asymmetries in PBF machines can produce gradients in build quality and defect populations across the build plate [18]. Due to this spatial variability, specimens were distributed randomly across the build surface with even groupings. This measure produces representative averaging within each group, minimizing skewing of the data due to specimen placement on the build plate. Groups SH-M, LTHP-M, and SB-M were fabricated with consistent manufacturer recommended build parameters and thus were averaged together. The averages of the above-mentioned metrics are reported for each group in Table 3.

### 2.3. HIP Treatment and Mechanical Testing

**Figure 2:**
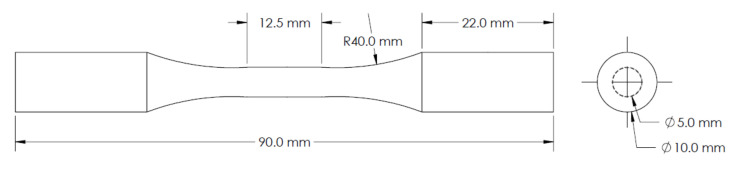
Fatigue specimen geometry.

After CT analysis, each group of specimens underwent their designated HIP treatments described in Table 2. HIP cycles were performed in an argon environment using a QIH-15L press HIP (Quintus Technologies AB, Vasteras, Sweden). Specimens were then machined into dog-bone geometries and surface finished in accordance with the ASTM-E466 standard for constant amplitude axial fatigue testing. The final geometry of the fatigue specimens can be viewed in Figure 2. Tension-tension fatigue tests were conducted in force control with a stress and frequency of 862 MPa and 10 Hz, respectively. All tests were performed with a load ratio of R = 0.1.

### 2.4. Microscopy

After fatigue testing, specimens from each group were selected for microstructure morphology characterization (i.e., average grain length and width). In order to consider the effect of build orientation on the anisotropy of AM microstructures, all specimens were sectioned from the gauge section in parallel (transverse) and perpendicular (normal) directions with respect to the build direction. Coupons were mounted and polished based on the ASTM-E3 standard. The mounted samples were mechanically ground using multiple sandpapers, from coarse grit size of 320 to fine grit size of 2500, and polished using 0.05 μm alumina polishing suspension until a mirror surface finish was achieved. After mechanical polishing, the samples were placed in a vibratory polisher with silica suspension for at least 6 h. The mirror-polished samples were put into a Crossbeam 550 Scanning Electron Microscope (SEM) (Zeiss, Oberkochen, Germany) to conduct electron backscatter diffraction (EBSD) analyses. All EBSD was processed under the same configurations (e.g., magnification of 200×, step size of 1 μm). If the zero-solution was greater than 10%, the samples underwent the above polishing procedure again to reduce the effects of noise. The post-processing of EBSD data was conducted by AztecCrystal from Oxford Instruments. To improve the quality of images, noise cancellation was applied once.

In addition to EBSD, specimens from each group underwent fractographic analysis. Fracture surfaces were analyzed using a VHX-6000 (Keyence International SA, Mechelen, Belgium). Initially, the entire surface was observed with low magnification to estimate the overall fracture surface. The higher magnification images were captured to locate distinct crack initiation sites and identify the main cause of failure.

## 3. Results and Discussion

To test the independence of HIP’d AM Ti-6Al-4V fatigue performance and initial material states, the ASTM recommended HIP (ASTM F2924 ∼920 ${}^{\circ }$C and 100 MPa for 2 h) was examined. Specifically, we show that the fatigue life of three different Ti-6Al-4V AM materials, subjected to the standard HIP and subsequent surface machining, can be significantly different. The three groups are labeled according to their HIP treatment (i.e., standard HIP—SH) followed by their level of initial defect densities (i.e., low (L), medium (M), or high (H) density), as will be discussed later in the manuscript. The first material (SH-M) was fabricated on the laser powder bed fusion (LPBF) DMP 320 (3D systems, Rock Hill, SC, USA) using manufacturer recommended build parameters for Ti-6Al-4V. The second (SH-H) was created on the LPBF system EOS M290 (EOS GmbH, Krailling, Germany), intentionally employing non-optimal build parameters such that build defects were ubiquitous throughout the specimens. The third (SH-L), was built on an M290 (EOS GmbH, Krailling, Germany) with manufacturer recommended build parameters, and had the lowest defect density of the three groups.

**Figure 3:**
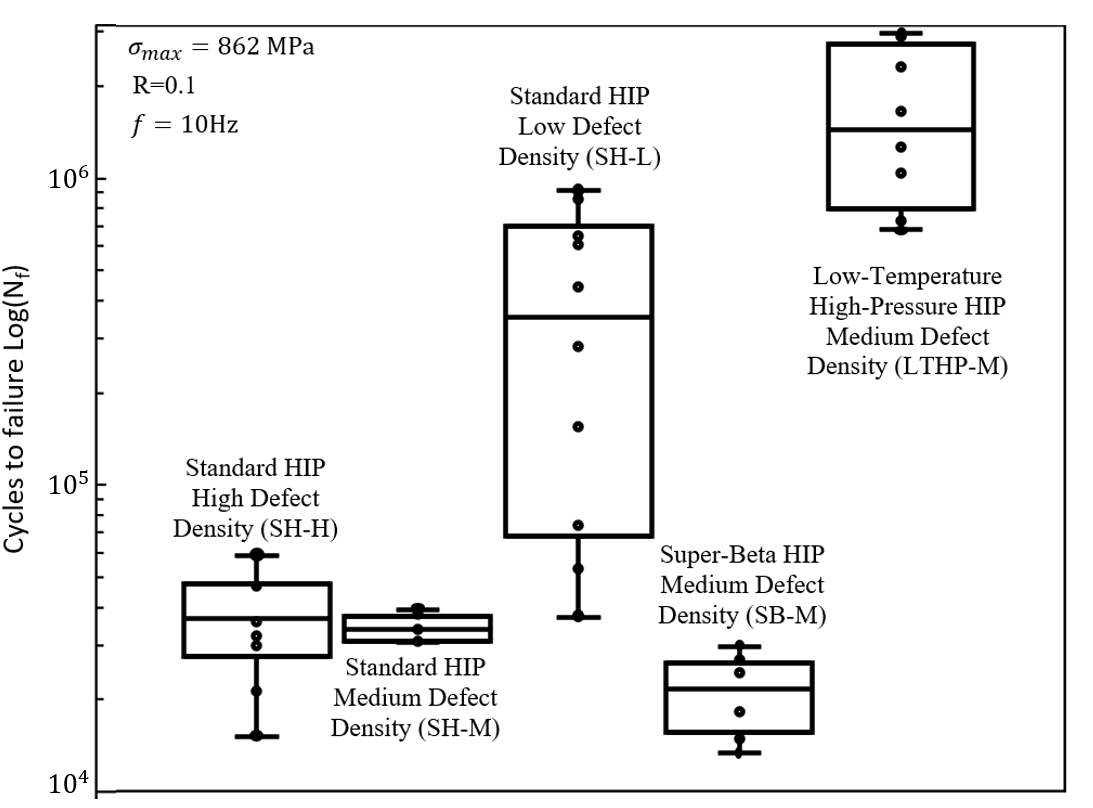
ASTM E-466 force controlled constant amplitude fatigue test results for five groups of AM Ti-6Al-4V specimens with different material states. The number of cycles to failure, $N_{f}$, for each specimen are represented by circles. The group’s lower, median, and upper quartiles are represented by the bottom, middle, and upper lines, respectively. The range of each data set is represented by the extended brackets. Each group’s defect density (low/medium/high) refers to the initial condition, before the application of HIP.

Fatigue test results are presented in Figure 3 where one case, group SH-L, exhibited an average fatigue performance approximately an order of magnitude greater than the other two standard HIP’d groups, i.e., 414,000 cycles vs. 34,000 cycles for group SH-M and 37,000 cycles for group SH-H. Large differences were also observed in performance variability, i.e., standard deviations of 3000 cycles, 13,000 cycles, and 355,000 cycles for groups SH-M, SH-H, and SH-L, respectively. While the number of specimens per test group was limited, the data is sufficient to confidently confirm a clear distinction in fatigue performance. A significant difference in fatigue response between group SH-L and groups SH-H and SH-M is established via the Kolmogorov0-Smirnov (K-S) statistic (*p* = $6.1\times 10^{-5}$). That is to say that despite these groups having undergone identical HIP treatments, the fatigue life varied significantly, i.e., fatigue performance is not independent of the material’s initial condition.

Next, it is established that the difference in fatigue performance between groups cannot be attributed to a disparity in commonly measured characteristics of the initial defect populations. Utilizing X-ray computed tomography (CT) analysis, the initial defect number density, porosity, and size were quantified for each group. As shown in Table 3, the initial defect porosity and number density in the standard HIP (SH) groups were found to differ by more than an order of magnitude. While the group with the greatest fatigue life (group SH-L) did have the lowest number density of initial defects, the inverse relationship was not present. More specifically, groups SH-H and SH-M had a difference in defect density of 3600%, yet their fatigue performances were quite similar. In group SH-H, the initial defect population not only encompassed far more defects, but it also contained a much larger average defect volume (142%), which some authors would suggest implies poorer fatigue performance (although for AM Ti-6Al-4V, a correlation between defect size and fatigue performance is not generally supported, as described in [22]). The above observations confirm our contention that these commonly observed initial defect characteristics do not govern post-HIP fatigue performance.

**Figure 4:**
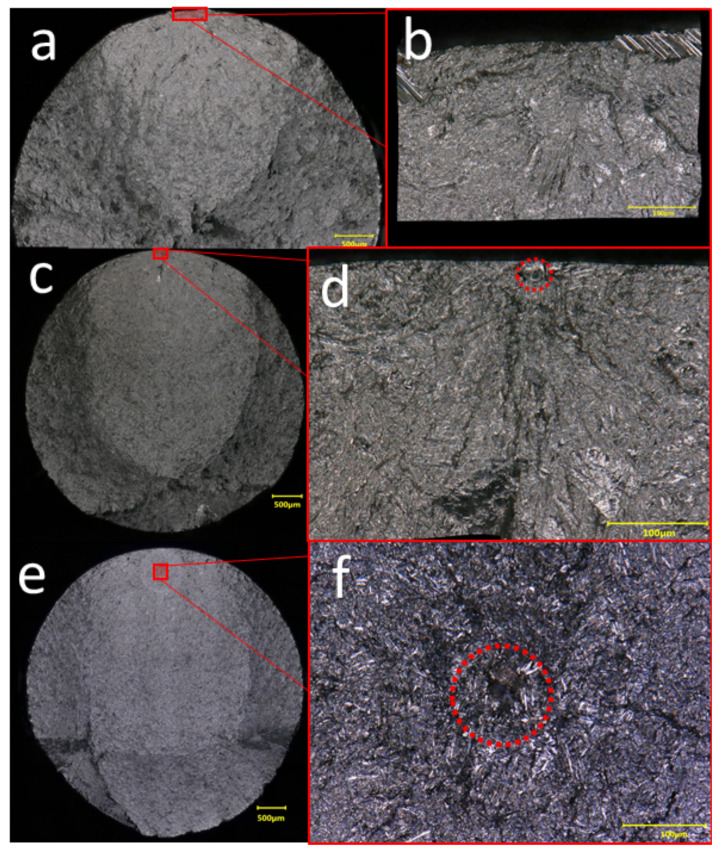
Two different fatigue crack initiation modes observed in AM Ti-6Al-4V having undergone standard HIP; (**a**,**b**) fracture surface of standard HIP’d specimen with medium initial defect density (SH-M) showing that failure initiated independent of build defects. Note in (**b**) there is no clear nucleation site. Fracture surface of (**c**,**d**) standard HIP’d specimen with low defect density (SH-L) and (**e**,**f**) high defect density (SH-H) where failure initiated from defects.

This assertion is consistent with our fractography analysis of the standard HIP’d (SH) groups, where fatigue failure initiation from, and independent of, internal defects is identified. Specifically, two instances of distinct failure mechanisms were observed. As shown in Figure 4, crack initiation from defects and independent of defects were both shown to be sources of failure in these groups. The observed crack initiation from HIP’d build defects is in direct contradiction to much of the literature, where HIP’d AM Ti-6Al-4V has been reported to fail only via crack initiation at microstructural features other than build defects [7,27,29,32,33,34]. In only a single other occasion are we aware of failure being reported to initiate from build defects after standard HIP, i.e., Molaei et al. [23]. In isolation, these findings might be taken to support an argument that post-HIP defect characteristics (including characteristics of the microstructure surrounding defects) solely influence post-HIP fatigue performance. However, this would not be consistent with both types of initiation sites observed; i.e., both crack initiation at defects and away from defects can lead to failure in HIP’d material. The observation of multiple types of initiation sites suggests that initiation site type does not necessarily govern the fatigue performance of HIP’d AM Ti-6Al-4V. There are two major implications of these results. The first implication is that models that utilize extreme value statistics to map defect characteristics to fatigue life [10,39,40] are not mechanistically supported for HIP’d Ti-6Al-4V. Importantly, this does not preclude the use of popular weakest link models for fatigue life prediction, so long as the “links” are associated with unspecified initiation sites, not necessarily build defects [41]. The second implication is that features other than defects must govern fatigue performance, i.e., properties of the microstructure.

**Figure 5:**
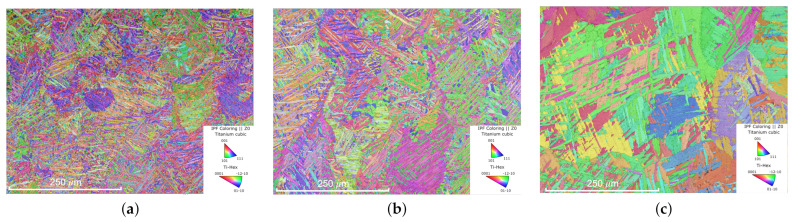
EBSD images showing the microstructure of specimens after HIP treatment from (**a**) a low-temperature high-pressure HIP group with high defect density (LTHP-M), (**b**) a standard HIP group with low defect density (SH-L), and (**c**) a super-beta HIP with medium defect density (SB-M). Note: both α and β phases are shown. The highest fraction of β of any group was ≤0.1% in the SB-M sample, as expected.

Having established that internal defects do not govern fatigue performance in HIP’d Ti-6Al-4V, the microstructures of each group were examined via Electron Backscatter Diffraction (EBSD) microscopy. Qualitatively, the microstructures morphology (i.e., average grain shape and orientation) of the groups that underwent standard HIP (SH) are indistinguishable. An example of an SH group microstructure is shown in Figure 5b. The results of quantitative image analysis produced very similar values between the SH group’s microstructure morphology, e.g., the average width and thickness of the primary α−laths shown in Table 4.

This result indicates that features of the microstructure beyond those traditionally characterized are responsible for the distinction in fatigue performance of the SH groups. Such features could come in two forms: (1) those that are not apparent via EBSD analysis such as characteristics of the pre-existing dislocation structure, grain boundaries, or chemical composition [42,43]; or (2) characteristics involving localized detrimental deviations from the average, e.g., sporadically occurring clusters of features or large alpha phases that would only appear by examining wider fields of view during microscopy. Features of both forms could influence both crack initiation and arrest. Recent work has shown that crack arrest can have a significant impact on the fatigue performance of metal AM materials [43,44,45]. These studies attribute the changes in fatigue performance primarily to increased crack arrest as a result of grain size affects. Due to the consistent average grain morphology observed here, the difference in fatigue performance cannot be attributed to the average morphology. In this sense, the average microstructure features (such as grain type, morphology, dislocation structure, etc.) may sufficiently arrest cracks, while local deviations from this average (form 2) possibly containing attributes not observed here (form 1), could be conducive to crack growth. For this reason, the average microstructure morphology is an insufficient indicator of fatigue performance in HIP’d AM Ti-6Al-4V.

**Figure 6:**
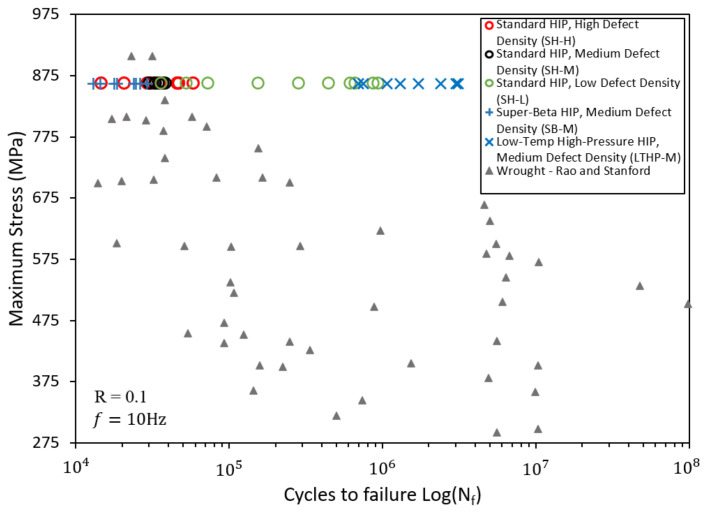
Fatigue life of all AM Ti-6Al-4V specimens tested in this work, with results for wrought Ti-6Al-4V aggregated by Rao and Stanford [30].

Having established that the prediction of HIP’d AM Ti-6Al-4V fatigue performance remains an open goal, it is useful to place this point in the context of traditional wrought behavior. Specifically, we compare the HIP’d AM fatigue data presented here to a wrought Ti-6Al-4V fatigue data set collected by Rao and Stanford [30] (Figure 6). Although statistically significant differences in fatigue performance existed between the standard HIP treated groups presented in Figure 1, on the whole, the standard HIP treated groups performed at the same level (groups SH-H/M) or far superior to (SH-L) wrought Ti-6Al-4V. We find this performance window to be impressive, considering that it resulted from such a wide variety of fabrication variables (e.g., AM system, build parameters, powder chemical composition, etc.).

While this work has established that the controlling features remain to be identified (and thus an ability to robustly predict the fatigue performance of HIP’d AM Ti-6Al-4V continues to be infeasible), we are able to report the existence of general trends consistent with well-established correlations. Specifically, when the temperature during HIP is increased above the ASTM standard recommended 925 ± 30 ${}^{\circ }$C, the fatigue performance decreases [21,46]. This was confirmed by testing an additional group of specimens (SB-M in Figure 6). This group, like the group SH-M, was fabricated on a DMP 320 (3D systems, Rock Hill, SC, USA) using manufacturer recommended build parameters. The specimens in SB-M underwent HIP at a super-beta soak temperature of 1050 ${}^{\circ }$C and a pressure of 100 MPa for 2 h (i.e., SB: super-beta HIP). Despite having a moderate amount of initial internal build defects, this group exhibited the worst fatigue life of any HIP treatment examined here (Figure 3), and performed worse when compared to wrought specimens (Figure 6). As shown in Table 4 and Figure 5c, group SB-M had an increased α-grain width by a factor of three and an increased length by a factor of almost two when compared to the standard HIP specimens. Consistent with traditional metallurgical wisdom on annealing temperature, the relatively poor performance of group SB-M is thus correlated to a coarsened microstructure resulting from the higher soak temperature HIP treatment (we note that the 1050 ${}^{\circ }$C treatment is above the beta transus temperature, and thus is distinctly different from the 920 ${}^{\circ }$C treatment, as it would completely erase any pinning alpha phases). That is to say that, although the controlling features have yet to be identified (as shown by the standard HIP results), when the grain morphology is massively increased, the influence of these unknown mechanisms (such as crack arrest) becomes overshadowed by the grain size effects on fatigue performance.

Alternatively, HIP at decreased temperatures would be expected to maintain a finer, more favorable microstructure, allowing features other than grain size to govern fatigue performance. In the case of AM Ti-6Al-4V, where initial defects are ubiquitous, the advantages of decreasing HIP temperature are expected to be negated by the detrimental effects of defects insufficiently minimized in the material. The authors hypothesized that this dilemma between optimal microstructure and defect minimization could be resolved by employing an HIP treatment at lower temperature and higher pressures (LTHP). Such a treatment would be capable of eliminating build defects as fatigue life-limiters, while the reduced temperature would maintain a fine microstructure. Applying the above rationale, the fatigue performance of specimens fabricated on the DMP 320 (3D systems, Rock Hill, SC, USA) subjected to HIP at 800 ${}^{\circ }$C and 200 MPa (i.e., group LTHP-M) were examined. Despite having relatively high levels of initial defects, the fatigue performance of this group, shown in Figure 3, far exceeds any group tested in this work, as well as the wrought Ti-6Al-4V presented in Figure 6. Table 4 and Figure 5a confirm that given this low-temperature/high-pressure treatment, the desired fine microstructure is preserved. With the LTHP-M HIP, the improved fatigue life is attributed to the adequate reduction of defects, while simultaneously minimizing grain growth such that other microstructure attributes govern performance. The authors suggest that future work should study the effects of this treatment on other mechanical properties beyond fatigue performance (i.e., strength, hardness, ductility).

## 4. Conclusions

In this study, the utility and limitations of ASTM recommended standard HIP of AM Ti-6Al-4V for fatigue critical applications were examined. This was achieved by fatigue testing several groups of HIP’d Ti-6Al-4V with varying initial material states. The material attributes which govern fatigue failure after HIP treatment were explored. Finally, the fatigue performance of alternative treatments to the ASTM standard HIP were tested. The significant findings resulting from this study are summarized below:

(1) The fatigue life of HIP’d Ti-6Al-4V is dependent on the initial material state. AM Ti-6Al-4V materials with different initial material states exhibited significantly different fatigue performance despite have undergone identical HIP treatment.
(2) Average microstructure morphology and defect population attributes do not govern fatigue performance in HIP’d AM Ti-6Al-4V. In direct contradiction to much of the literature, where defects were reportedly neutralized by HIP, crack initiation was observed both at and independent of defects. This suggests that initiation site type does not control failure. Popular models that employ extreme value statistics to map defect characteristics to fatigue performance are therefore not mechanistically supported for HIP’d Ti-6Al-4V.
(3) Commonly examined material attributes (e.g., defect size/shape, and average grain morphology) were insufficient to explain the observed discrepancy in fatigue performance. This result implies that attributes beyond those commonly examined in AM materials, or local deviations from these average attributes, should be considered as mechanisms for both crack initiation and arrest.
(4) Despite a relatively high defect density, a low-temperature high-pressure HIP treatment produced fatigue performance far superior to the ASTM recommended standard HIP. It is recommended that this new treatment be applied to fatigue critical applications.

## Figures and Tables

**Table 1:** Ti-6Al-4V infill build parameters fabricated on the M290 and DMP320 LPBF systems. Note: Group SH-L was fabricated with reduced power to intentionally produce components with higher defect populations.

Group	Laser Power (W)	Scan Speed (mm/s)	Hatch Distance (µm)	Layer Thickness (µm)	Beam Diameter (µm)	Energy Density (J/mm^3^)
DMP:SH-M,SB-M,LTHP-M	245	1250	82	60	85	39.8
EOS:SH-H	160	1200	140	30	100	31.7
EOS:SH-L 3	280	1200	140	30	100	55.6

**Table 2:** HIP treatment parameters for each group examined in this study.

Group	Description	Temperature (${}^{\circ }$C)	Pressure (MPa)	Soak Time (h)
SH	Standard HIP	920	100	2
SB	Super-Beta HIP	1050	100	2
LTHP	Low-T/High-P	800	200	2

**Table 3:** Quantification of the initial defect populations measured via X-ray CT. Note: groups SH-M, SB-M, and LTHP-M were taken from the same build using identical fabrication parameters, and thus contained similar average defect distributions.

HIP Treatment	Defect Level	Defect Diameter	Defect Density	Porosity
Group Label	(H/M/L)	(μm)	(Defects/mm${}^{3}$)	(%)
Standard HIP (SH-H)	High	58.7	265.85	3.96
Standard Hip (SH-M)				
Low-Temperature High-Pressure (LTHP-M)	Medium	41.2	7.27	0.036
Super-Beta (SB-M)				
Standard HIP (SH-L)	Low	49.3	0.35	0.00264

**Table 4:** Quantified average α-lath length and width after HIP. Note: defect level refers to the relative defect density for each group quantified via CT analysis prior to HIP.

Group Label	SH-H	SH-M	SH-L	SB-M	LTHP-M
HIP Treatment	Standard HIP	Standard HIP	Standard HIP	Super-Beta HIP	Low-Temp High-Pressure HIP
Defect Level	High	Medium	Low	Medium	Medium
Length (μm)	45.0	49.5	46.4	85.3	40.4
Width (μm)	4.3	4.4	4.2	12.9	3.1

## Data Availability

Not applicable.
